# Chemomechanical damage prediction from phase-field simulation video sequences using a deep-learning-based methodology

**DOI:** 10.1016/j.isci.2024.110822

**Published:** 2024-08-26

**Authors:** Quan Zeng, Shahed Rezaei, Luis Carrillo, Rachel Davidson, Bai-Xiang Xu, Sarbajit Banerjee, Yu Ding

**Affiliations:** 1 H. Milton Stewart School of Industrial and Systems Engineering, Georgia Institute of Technology, Atlanta, GA 30332, USA; 2 Institute of Materials Science, Mechanics of Functional Materials, Technische Universität Darmstadt, 64287 Darmstadt, Germany; 3 Department of Chemistry, Texas A&M University, College Station, TX 77843, USA; 4 Department of Chemistry and Biochemistry, University of Delaware, Newark, DE 19716, USA; 5 Department of Materials Science and Engineering, Texas A&M University, College Station, TX 77843, USA

**Keywords:** Chemistry, Physics, Computer science

## Abstract

Understanding the failure mechanisms of lithium-ion batteries is essential for their greater adoption in diverse formats. Operando X-ray and electron microscopy enable the evaluation of concentration, phase, and stress heterogeneities in electrode architectures. Phase-field models are commonly used to capture multi-physics coupling including the interplay between electrochemistry and mechanics. However, very little has been explored regarding developing predictive models that would forecast imminent failure. This study explores the application of convolutional long short-term memory networks for damage prediction in cathode materials using video sequence from phase-field simulations as a proxy for video microscopy. Two models are examined making use of, respectively, the damage video only and the damage and hydrostatic stress videos combined. We use customized quantitative metrics to compare the performance of the models. Our work demonstrates the outstanding capability of deep learning models using limited data to predict fracture behavior of battery materials, including crack propagation angle and length.

## Highlights

• Deep-learning-based method to predict chemo-mechanical processes in electrode materials
• Prognostication of crack development and propagation based on machine learning
• Potential to integrate in battery management systems of large-format batteries

## Graphical abstract

**Figure:**
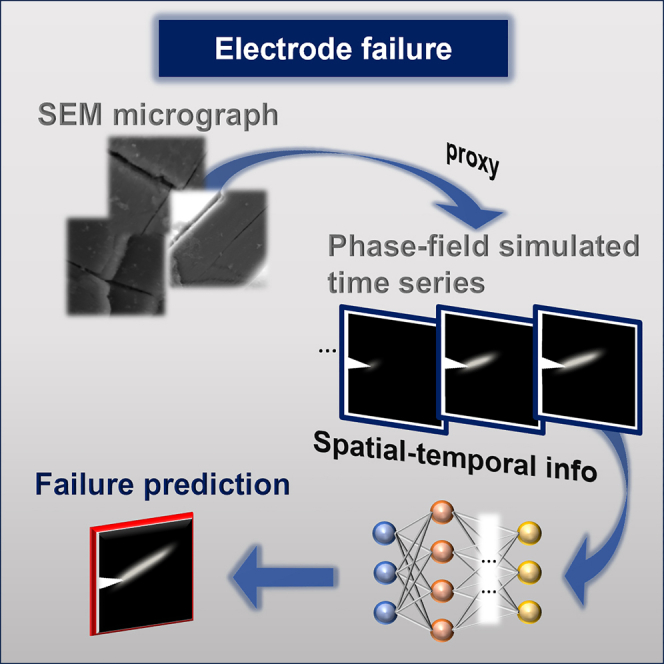

## Introduction

The loss of capacity of Li-ion batteries upon prolonged cycling is traceable in large measure to degradation mechanisms that arise from the coupling of electrochemistry and mechanics.^1^^,^^2^^,^^3^^,^^4^ Compositional gradients arising from inhomogeneities of the Li-ion diffusion flux and its propagation through an insertion host result in local stress gradients,^5^ which are exacerbated in phase-transforming materials by intercalation-induced structural transformations. Structural transformations induce elastic misfit and lattice coherency strains or give rise to dislocations^6^^,^^7^ at semi-coherent interfaces. In brittle oxide intercalation hosts, stress accumulation upon continuous electrochemical cycling engenders intergranular or transgranular fracture and/or decohesion of particles from the bulk electrode or current collector, resulting in an irreversible loss of capacity. Therefore, deciphering mechanisms of inelastic deformation and fracture is a key imperative to extending the lifetime of Li-ion batteries.^8^

One efficient method for studying fracture behavior is to simulate the process using phase-field (PF) fracture and related finite element simulations.^9^^,^^10^ PF damage formulations show great potential in predicting crack nucleation, propagation, and branching in various complex multi-physics problems. PF fracture models rely on introducing a length-scale parameter^11^^,^^12^ and by manipulating the gradient term in the formulation, can successfully capture anisotropic crack propagation.^13^ Wu and Nguyen^14^ proposed a new PF fracture model, which takes into account the cohesive nature of the fracture, where in addition to the fracture energy, the material ultimate strength is also a direct input for the model. Such models are essentially length-scale independent, which makes them promising candidates for capturing the full complexity of battery electrodes.^10^ PF models have been successfully applied in the context of chemo-mechanical fracture in battery systems. Through various contributions such as Miehe et al.,^15^ Zhang et al.,^16^ Zuo and Zhao,^17^ Klinsmann et al.,^18^ and Xu et al.,^19^ PF fracture models have been adapted to study crack formation across lithiation and/or delithiation cycles including inter- and intra-granular damage modes.^10^^,^^20^

Despite their great potential, PF models remain computationally expensive and difficult to execute in batchwise format since oftentimes sharp and arbitrarily complicated gradients need to be accurately computed across the system. Therefore, new techniques at different levels of computation are required to speed up the process. Machine learning (ML) methods have emerged as powerful tools to facilitate predictive design and to accelerate numerical modeling with reduced computational effort and greater generalization ability.^21^ Montes de Oca Zapiain et al.^22^ proposed a surrogate model that learns the microstructural evolution of targeted systems by combining statistically representative, low-dimensional description of the PF data and history-dependent ML techniques. The high-dimensional microstructural representation given by the microstructure autocorrelations is simplified though principal component analysis and then modeled using a long short-term memory (LSTM) neural network to accelerate the PF framework. Alhada-Lahbabi et al.^23^ presented a neural-network-trained model, which includes supervised and nonsupervised learning of Landau energy landscapes for ferroelectric PF modeling and predicts the polarization field evolution in the microstructure determining the electrostatic and mechanical equilibrium at each time step.

In this article, we create an ML video processing model to predict crack formation, gain a deeper understanding of the failure process, and provide a means of early failure detection through approximation of a segment of a multi-physics PF simulation video sequence. It is worth nothing that our purpose is not to build a computation-accelerating surrogate model to replace or complement the PF simulations, but instead to treat the PF output as realistic proxies of physical microscopy video data. In this context, the PF simulation introduces a pre-notch or pre-crack to emulate crack nucleation arising from the presence of surface defects. Our purpose is to develop a method that could be applicable, should the *operando* optical, scanning transmission X-ray, or electron microscopy video sequences that afford clear contrast mechanisms for imaging fracture become available such as to enable real-time battery control. The key findings reported in this paper thus correspond to video prediction rather than the improvement of surrogate models of PF simulations. The design of the model structure developed here does not explicitly encode any specific physics domain knowledge derived from partial differential equations used in the PF simulations nor does it adapt to the shape of any initial notch if there is one. The predictions of crack initiation and propagation are made based on the damage field and stress field simulation rather than a specific geometry. We instead examine the ability of the model to reveal the spatial-temporal evolution of inelastic deformation and fracture.

**Figure 1:**
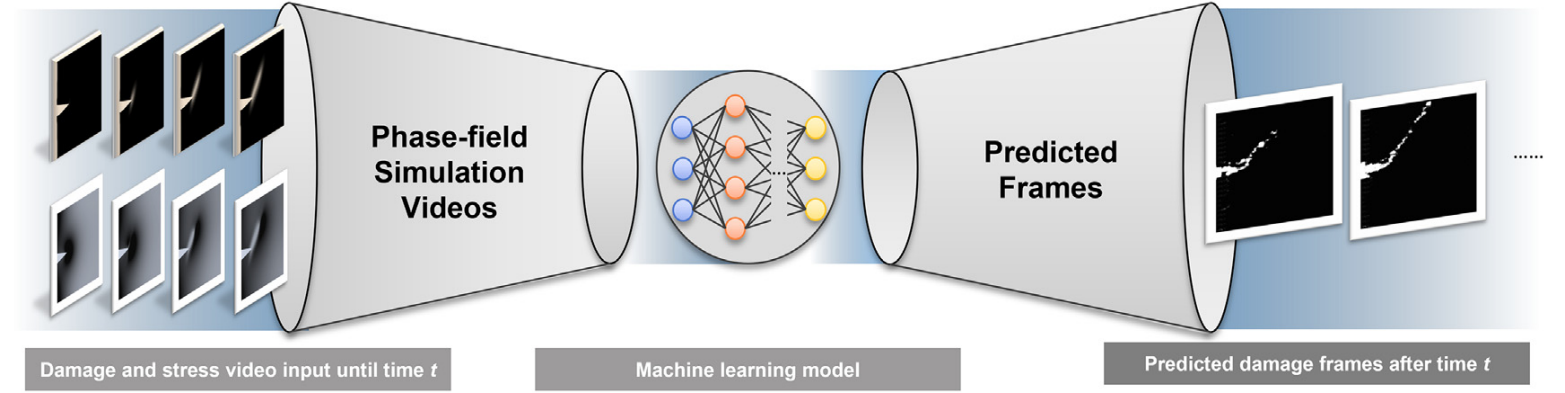
Overall workflow The machine learning model replaces the simulation model by generating image frames after time t. A ConvLSTM neural network takes the damage and stress video frames simulated by a PF simulation as inputs and predicts damage progress in future video frames. The deep learning model provides a means of accelerated failure detection based on the previous sequence of dynamic time-correlated images.

Our model is a deep learning (DL) model, which arguably is among the most common approaches for learning features directly from raw video/image data. Hochreiter and Schmidhuber^24^ proposed a recurrent neural network with feedback connections—the LSTM network—which has been increasingly used to solve the time-series prediction problem. To learn good video representations, Srivastava et al.^25^ used a composite model consisting of an autoencoder and a future predictor based on LSTMs. Lew et al.^26^ applied a ConvLSTM-based model to physics-based molecular modeling (MD) simulations to learn the spatiotemporal relations of crack propagation. Wang et al.^27^ developed a DL model, StressNet, to predict the sequence of maximum internal stress by combining a temporal-independent convolutional neural network and bi-directional LSTM. Despite recent advances, the use of DL models with multi-source data to predict the propagation of fracture patterns in materials remains limited. In this paper, we report our effort that builds a ConvLSTM neural network to predict damage initiation and propagation using the damage information along with internal stress information output from the PF simulations; see Figure 1.

## Results

### Crack formation in lithiation process

**Figure 2:**
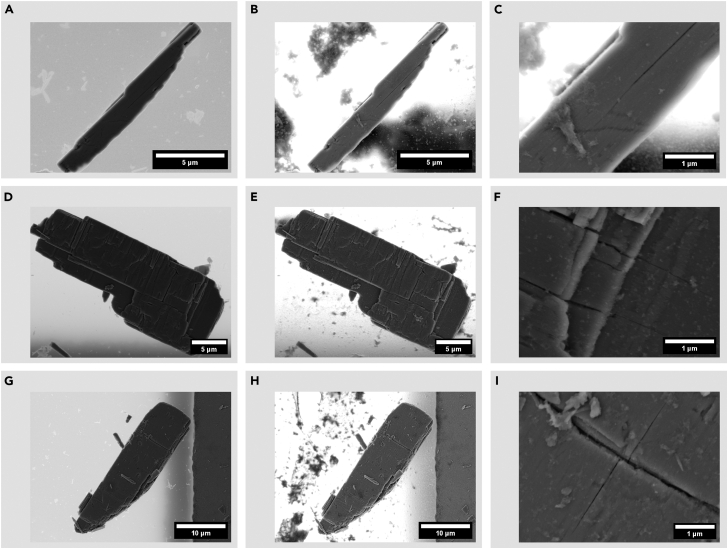
SEM images of crack formation in lithiated α-V_2_O_5_ single crystals (A–I) SEM images of exfoliated single crystals that underwent three chemical lithiation and three delithiation cycles where (A–C) depict crack elongation, (D–F) depict crack formation along stair-step layers, and (G–I) show perpendicular secondary crack formation.

Crack nucleation and growth phenomena can follow a wide variety of patterns, as exemplified by experimental data shown in Figure 2 for a single crystal of a canonical intercalation host, α-V_2_O_5_. Three lithiation/delithiation cycles led to crack expansion as well as new secondary crack formations branching from previously formed cracks present before lithiation in α-V_2_O_5_. Cracks present before lithiation provide a means for the Li-ion flux to engender local lattice expansion and contractions, which results in crack propagation and formation of secondary cracks, exposing new surfaces for interaction with the electrolyte (Figures 2A–2C). We note that crack propagation occurred perpendicular through stair-step layers due to increased flux during lithiation/delithiation processes (Figures 2D–2F) as seen before for another 2D-layered insertion host γ′-V_2_O_5_.^28^ Similarly, lithiation-induced flux across crack formations formed from deintercalation processes can lead to crack formations post-lithiation perpendicular to the pre-lithiation crack (Figures 2G–2I). Such lithiation-induced cracks form due to the brittle nature of V_2_O_5_ where lattice expansion and contraction especially across phase boundaries lead to elastic misfit and crack formation/propagation.

### PF model simulations

**Figure 3:**
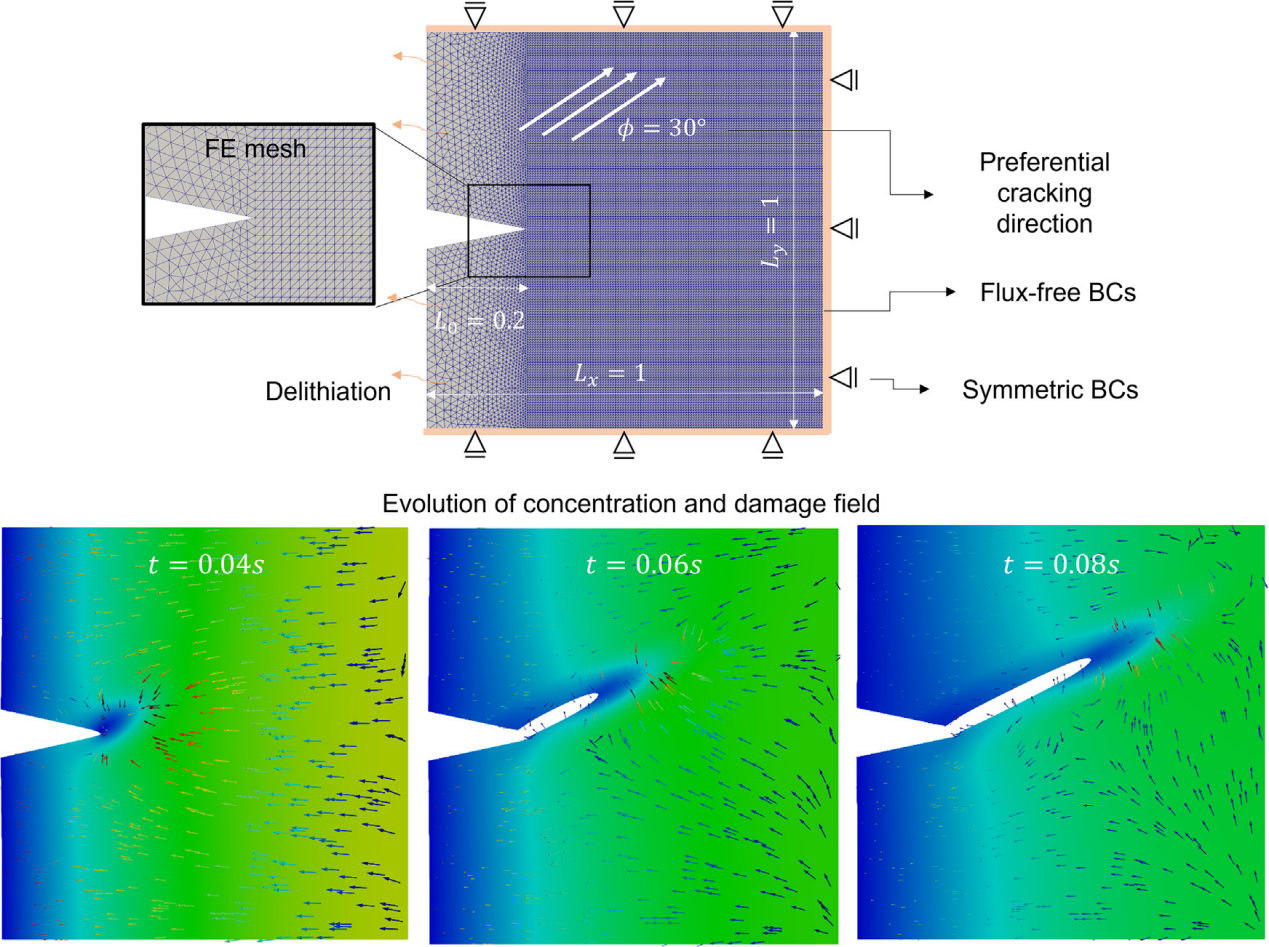
Simulating crack propagation for ϕ = 30° Top: geometry and finite element mesh for the simulation. Bottom: results of the multi-physics finite element calculation in terms of concentration and damage field through time.

Physically meaningful connections between flux, displacement, and stress fields are crucial in the context of computer modeling of fracture in chemo-mechanical systems. Therefore, we investigate a case where change in chemical flux drives damage progression and three main fields—displacement field, concentration field, and damage field—are connected together in a chemo-mechanical coupled environment. More detailed formulation and parametrization are provided in the study by Rezaei et al.^10^ The geometry and the boundary conditions are according to Figure 3. The initial concentration of c_0_ = 0.9c_max_ is kept within the bulk. The concentration on the left edge is kept constant by applying a Dirichlet boundary condition (c_min_ = 0.1c_max_). All other surfaces are insulated.

Dependent on the microstructural features or atomistic direction within the structure of a crystal, there might exist some preferential direction for the crack. The latter is also known as the cleavage plane and can serve as a potential site for the initiation of cracks. Figure 3 presents one example where an angle of ф = 30° is used as the preferential crack direction. Upon delithiation, the crack tends to deviate from the horizontal line. In other words, the crack tends to propagate along the weakest direction. Furthermore, we set the parameter α = 10 for this simulation (see Equation 7). The output of the PF simulation is a series of video frames, which are reminiscent of physical videos obtained in field through *operando* video microscopy. These simulation video frames are the *only information* used as inputs to the subsequent ML models.

### Damage prediction using DL models

#### Problem formulation

We propose an ML method as a possible alternative to computationally more expensive finite element simulations for facilitating real-time battery control. As mentioned earlier, the simulation videos are treated as realistic proxies and can in principle be supplanted by physical microscopy video from any imaging mechanism that differentiates fractured regions from the substrate.^29^

Given a sequence of image frames from a PF simulation video, {X_1_, X_2_, …, X_T_}, we aspire to predict the future H image frames, {$\hat{X}$_T+1_, $\hat{X}$ _T+2_, …, $\hat{X}$_T+H_} and identify cracks and other anomalies in a post hoc manner. One can use ML to learn a function that maps a sequence of input images to an output image or a sequence of output images given in Equation 1.

(Equation 1) $${\hat{X}}_{T+h}=f\left(X_{1},X_{2},\ldots ,X_{T}\right),h=1,\ldots ,H$$

where *f* is the mapping function.

We employ two different DL models for the given purpose. The first model, the damage model, is based on Srivastava et al.^25^ and uses only the historical damage profile to predict the future damage image frame. The second model, the ensemble model and a modification of Wang et al.,^27^ uses both the historical damage and stress profiles to predict the future damage image. For both models, we constrain ourselves to employ four frames, d = 4, to make one-step ahead prediction at h = 1.

#### Data description and preprocessing

In this study, we use the PF fracture simulations to generate eight videos of stress and damage profiles of a square-shaped sample with a notched left edge. Each video displays a distinct grain orientation. The eight grain orientations are 0°, 10°, 25°, 30°, 45°, 60°, 75°, and 90°, respectively.

The resolution of the original image is 792 × 1216, which is relatively high for training convolutional neural networks (CNNs). Image resolutions used for training CNNs are generally between 64 × 64 and 256 × 256.^30^ To reduce the risk of model overfitting, it is often desirable in applications of deep architectures to minimize the number of input variables or features that must be optimized.^31^ Therefore, we preprocess both stress and damage videos by cropping the surrounding white space and downsampling the cropped images to a resolution of 224 × 224.

**Figure 4:**
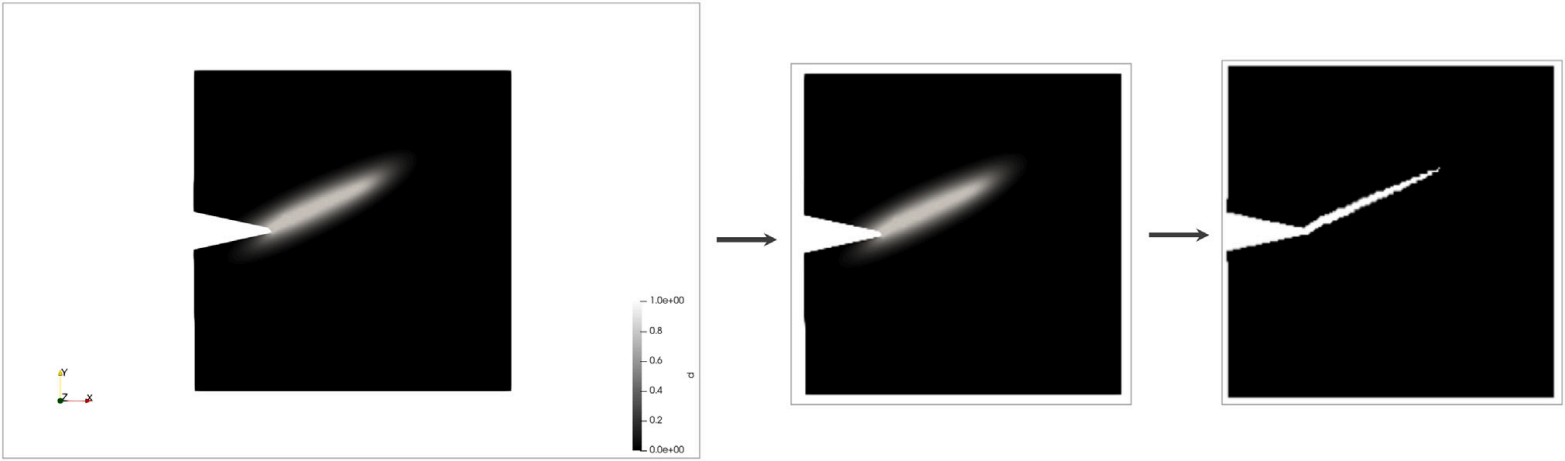
Video image preprocessing Left: original damage frame at 30°. Middle: cropped and downsampled frame. Right: binarized frame.

Existing image preprocessing strategies^32^^,^^33^ advocate the use of binarization for making the path of the cracks in the damage videos sharper. The downside is that after binarization, some useful information in the original grayscale images is possibly lost. We have explored both options and contrasted the performance of the ensemble model with and without image binarization. Binarization is achieved by converting the damage frames from their original grayscale to binary images. The threshold used for binarization is 0.75, i.e., when a value greater than 0.75 is set to 1 and a value smaller set to 0. Figure 4 shows the cropping, downsampling, and binarization of the damage video image.

#### Training and testing settings

Since each PF simulated video has a preferential direction for the crack, we split the training and testing data based on the distinct grain orientation. Seven out of the eight grain orientations videos are used for training, whereas the eighth grain orientation, which has not been used in training, serves as the holdout set for out-of-sample testing. The test set is then an indicator of overfitting, enabling evaluation of whether the model can be generalized to a new, unseen grain orientation. In the numerical study, we conduct two training-test rounds, which use the 0° video and the 60° video as the test set, respectively. Specifically, in the round when the 0° video is used for testing, the videos of grain orientations of 10°, 25°, 30°, 45°, 60°, 75°, and 90° (without 0°) are used for training, whereas when the 60° video is used as the test set, the videos of grain orientations of 0°, 10°, 25°, 30°, 45°, 75°, and 90° (without 60°) are used for training.

The videos contain a different number of image frames, ranging from 31 to 48, and in general only 6 frames capture the initiation and development of cracks. Therefore, to strike a good balance between maintaining a reasonable number of training samples and keeping some unused image samples for testing, we divide each video into sub-videos of five frames in order to efficiently train and test the model. Overextending the length of each sub-video would lead to a decrease in the number of training samples, whereas too short sub-video may not contain sufficient details to recover the underlying dynamics of the process. Suppose that a video has 35 frames, with each frame labeled as #1, #2, …, #35, respectively. The sub-video sequences, each containing five frames, are grouped as such: #1, #2, …, #5, #2, #3, …, #6, …, #31, #32, …, #35. As we aim to make a one-step ahead prediction, for each sub-video sequence, the first four frames are used as training input and the fifth frame is used as training output. For example, for the first sub-video sequence, #1, #2, #3, #4 are used as input to predict the appearance of frame #5. Next, the summation of the squared difference between the predicted fifth frames and the actual fifth frames are used to tune the parameters in the model. In total, we have 260 input-output pairs for training and 30 pairs for testing in the first training-test round, and 263 pairs for training and 27 pairs for testing in the second round.

#### Damage prediction

**Figure 5:**
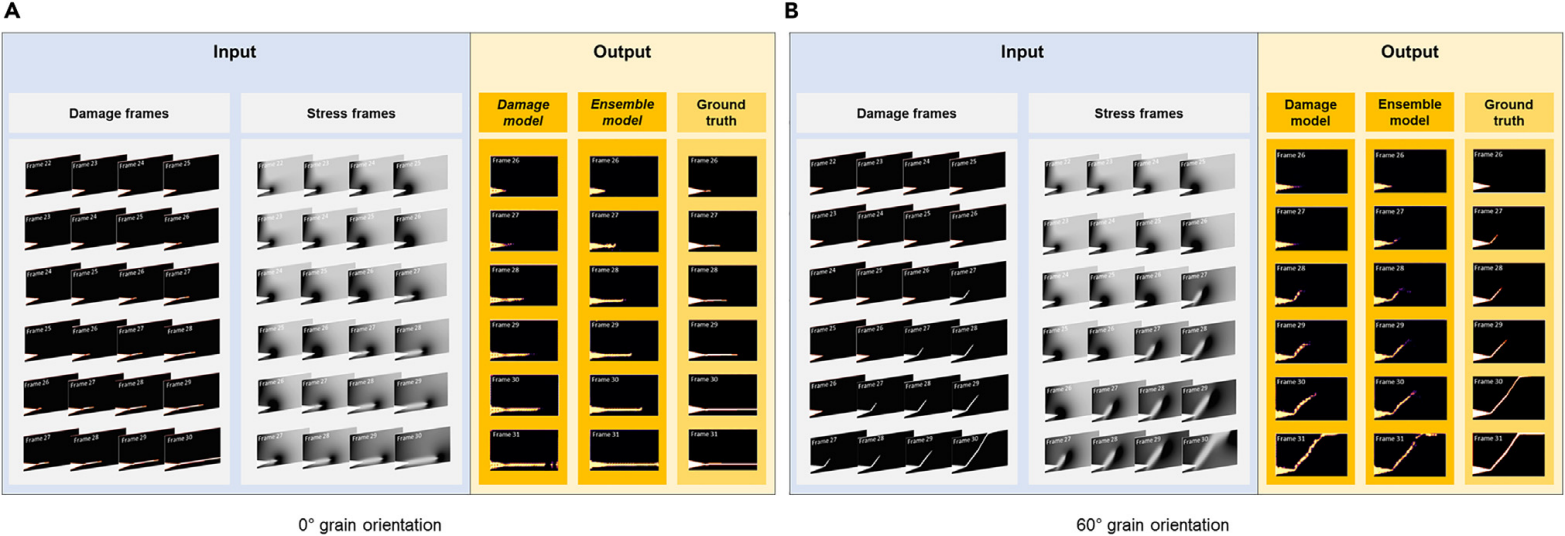
The test results of the damage and ensemble model using binary data (A) 0° grain orientation, (B) 60° grain orientation. The first four frames of each sub-video are used as input. The damage profile is used by both the damage model and the ensemble model, whereas the stress profile is used only by the ensemble model. The output is the predicted damage frame.

Let us first visually inspect the results to gain an intuitive understanding of the performance of the two different DL models. Figure 5 shows the output of the damage and ensemble models at 0° and 60° grain orientations, respectively. At frame #26 and #27 for 0° and frame #27 for 60°, the predicted crack growth output by the damage model alone apparently lags behind the actual growth. Adding the stress profile to the model yields more promising results. The lag between the prediction and the actual frame becomes smaller. For instance, the predicted fracture path at frame #27 for 0° grain orientation is longer and more distinct than what the damage-alone model predicts. For the 60° grain orientation, the ensemble model is even able to predict the correct grain orientation at frame #27.

**Figure 6:**
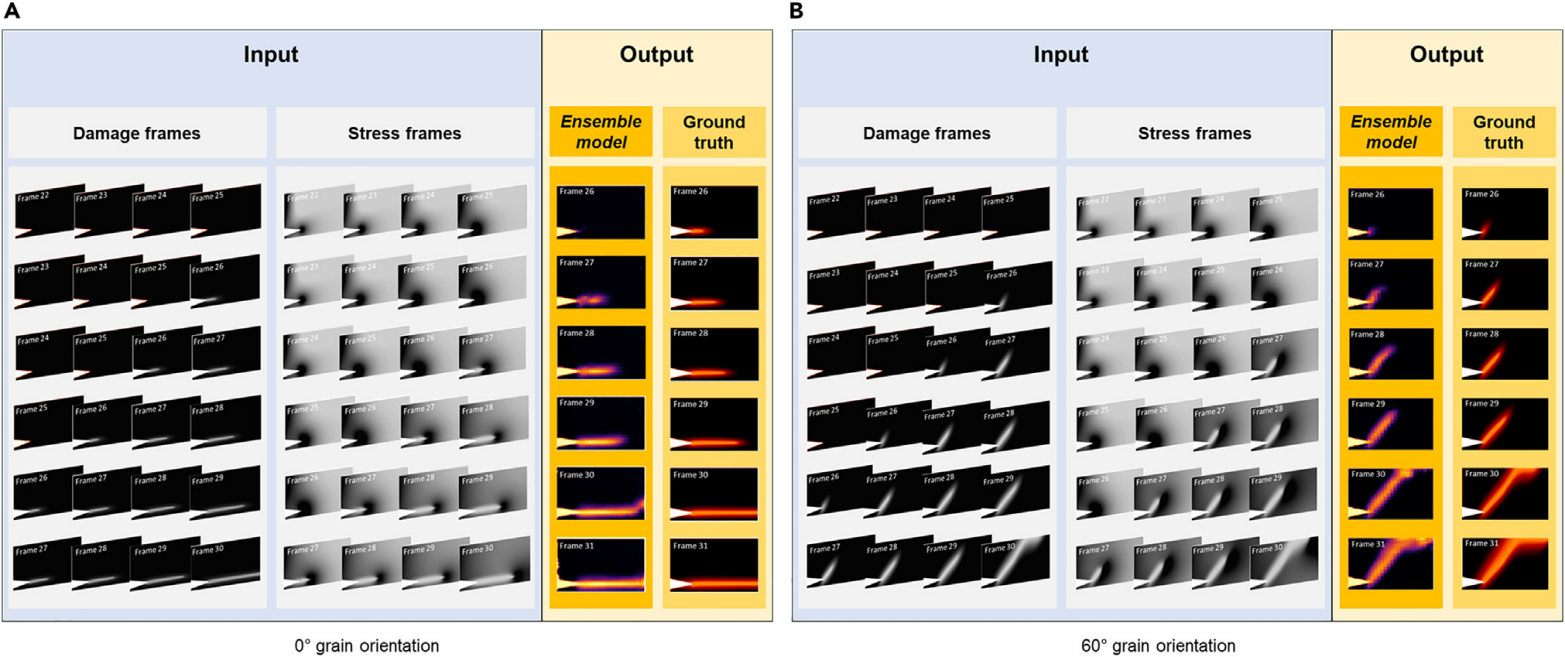
The test results of the ensemble model using original data (A) 0° grain orientation, the lag between predicted crack growth and the truth is further decreased. (B) 60° grain orientation, the model is able to predict crack initiation as soon as the true crack happens.

Considering that binarization may leave out useful information contained in the original grayscale images, we also conduct experiments using the original images and the ensemble model. The outcomes are presented in Figure 6. We do observe differences in the initial stages of crack propagation, when using the binary damage images and the grayscale images, indicating that the early crack path is indeed masked by binarization. After removing the threshold, the damage profile more effectively contributes to the prediction—the lengths of the predicted cracks from frame #27 onward for both grain orientations are longer than in the ensemble model using the binary damage input. In general, the predicted cracks for both degrees closely match the prevailing pattern, despite the minor variations in hues.

#### Performance evaluation

Next, we quantify the quality of the damage prediction for each model using the following four evaluation metrics: the initiation lag, crack length ratio, crack width ratio, and crack angle deviation. We are purposeful in selecting these rather than generic image quality metrics such as peak signal-to-noise ratio (PSNR)^34^ and structural similarity index measure (SSIM).^35^ The reason for our use of the customized metrics is because our objective here is to evaluate the local crack path prediction, instead of image reconstruction or enhancement. In other words, PSNR and SSIM measure if the processed images are clearer than the raw images. In our context, the image predicted by our model for time *t*, if compared with the raw image at time *t*, is always less clear—our image is a prediction and the raw image is the ground truth, so much so, that it is not practical to expect the prediction to be as good as the ground truth, much less so to demand the prediction to be better. As such, PSNR and SSIM are not good measures of the quality of prediction relevant to our objectives.

**Figure 7:**
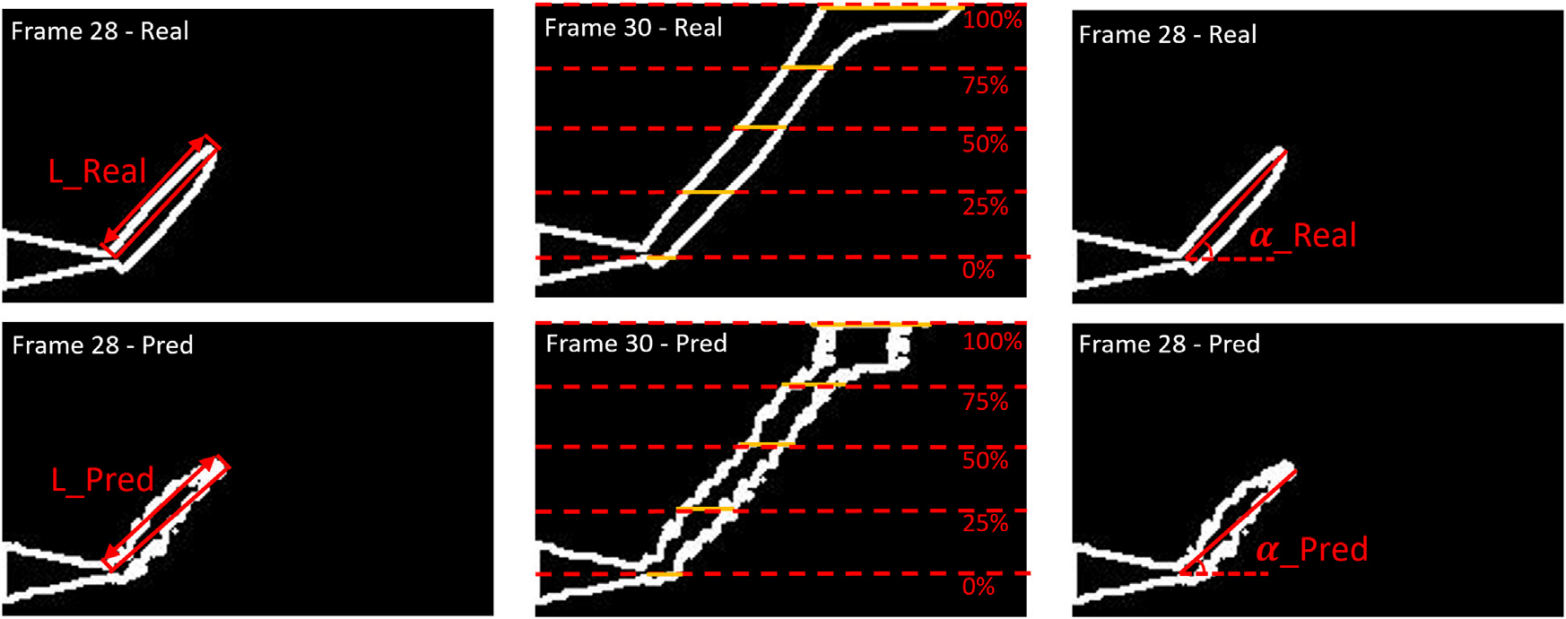
Illustration of the quantitative performance measures Left: the crack length measurements. Middle: the crack width measurements. Right: the crack angle measurements. All measurements are displayed for both the real frame and predicted frame at 60° grain orientation.

In the context of local crack path prediction, the key frame is the frame in which crack initiation or propagation occurs. Notably, the predicted frame images are always in grayscale, even if binary damage images are used as training inputs. The crack boundaries on grayscale images are harder to define, as they gradually fade into the background. When using binary damage images as inputs, the predicted cracks have sharper boundaries (albeit in grayscale), whereas when using original gray images, the predicted cracks have broader, fuzzier boundaries. In order to render a fair comparison between models and when using distinct data types, we apply the binarization process again to all the predicted damage images using a threshold of 0.6, so that the crack boundary is clearly and consistently defined. The final results are shown in Figure 7, where all the metrics are then calculated based on the measurements taken on the white contours. The initiation lag is computed by comparing the image frame index difference between the actual and predicted frames in which the crack first appears. The crack length is measured from the notch tip to the crack tip. The five crack widths are measured at 0%, 25%, 50%, 75%, and 100% of the total length, along the path of the crack. The crack angle is calculated using the arc-tangent function, and the absolute difference between the predicted crack angle and the actual one is recorded as the crack angle deviation.

We further calculate a crack length ratio using Equation 2, a crack width ratio using Equation 3, and the crack angle deviation using Equation 4. Because we measure five crack widths along the path of the crack, we use the median value to generate a single crack width ratio per frame for easy comparison.

(Equation 2) $$Crack\;Width\;Ratio=\frac{Median\;Predicted\;Crack\;Width}{Actual\;Crack\;Width}$$

(Equation 3) $$Crack\;Length\;Ratio=\frac{Median\;Predicted\;Crack\;Length}{Actual\;Crack\;Length}$$

(Equation 4) CrackAngleDeviation=|ActualCrackAngle−PredictedCrackAngle|

We calculate the metric values for both the damage and ensemble models using the binary damage images, as well as for the ensemble model using the original grayscale damage images. Table 1 records four performance metrics. What is reported therein is the initiation lag, corresponding to the video sequence in which the crack first appears, and the medians of the other three metrics over the entire training/test sub-video sequence pairs.

**Table 1:** Numeric test results

0° Grain orientation		Frame 26	Frame 27	Frame 28	Frame 29	Frame 30	Frame 31	Median
**Damage model – binary images**	**LR**	0	0.471	0.781	0.846	0.656	1.000	0.719
	**WR**	0	0.333	1.467	1.500	1.286	1.429	1.357
	**AD**	0	2.384	1.005	0	0	0	0
	**Lag**	1	–	–	–	–	–	–
**Ensemble model – binary images**	**LR**	0	0.673	0.900	0.794	0.706	1.006	0.750
	**WR**	0	1.000	1.467	1.333	1.286	1.333	1.309
	**AD**	0	3.464	1.818	1.414	1.014	0.712	1.214
	**Lag**	1	–	–	–	–	–	–
**Ensemble model – original images**	**LR**	0	0.915	0.963	0.885	1.006	1.063	0.939
	**WR**	0	0.854	0.800	1.333	1.200	1.321	1.027
	**AD**	0	2.027	1.438	0.504	0.358	0.358	0.431
	**Lag**	1	–	–	–	–	–	–

**60° Grain orientation**		**Frame 26**	**Frame 27**	**Frame 28**	**Frame 29**	**Frame 30**	**Frame 31**	**Median**

**Damage model – binary images**	**LR**	10.548	0.190	1.166	0.854	0.524	0.924	0.889
	**WR**	10.000	0.438	1.389	1.444	1.300	0.901	1.344
	**AD**	5.427	36.758	6.737	5.480	1.504	2.015	5.453
	**Lag**	0	–	–	–	–	–	–
**Ensemble model – binary images**	**LR**	2.062	0.673	1.269	0.990	0.594	0.897	0.944
	**WR**	2.000	1.000	0.500	0.944	0.286	0.900	0.922
	**AD**	13.765	12.993	17.311	0.377	1.532	3.180	8.087
	**Lag**	0	–	–	–	–	–	–
**Ensemble model – original** **Images**	**LR**	0.779	0.537	1.047	1.018	0.945	1.009	0.977
	**WR**	1.200	0.727	1.257	1.214	1.091	1.010	1.145
	**AD**	25.413	19.289	3.718	2.410	1.801	0.246	3.064
	**Lag**	0	–	–	–	–	–	–

LR, WR, and AD stand for the crack length ratio, crack width ratio, and crack angle deviation, respectively. Lag stands for the initiation lag. Lag is only reported at the initial key frame.

The numerical results are consistent with visual inspection, especially in terms of length and width ratios. The ensemble model predicts the length and width of the actual crack with increased accuracy and consistency. Our model performs well in predicting the crack direction for 0°, in which the angle deviation values are less than 2°. On the other hand, the prediction for the 60° crack propagation has a larger, more noticeable angle deviation, as a result of noise in the predicted frames, an issue that needs to be addressed in future research. The ensemble model with the original images performs better than the ensemble model with binary data. In most instances, the length and width ratios derived from the original data are more stable and closer to one. Intriguingly, when using binary images, the sharpness of the predicted cracks, in terms of the width ratio, is slightly better than using gray images for the 60° grain orientation case. This is explicable, because as stated earlier, using binary images as training input does render a sharper crack boundary in prediction. But the advantage is not significant, and such an advantage does not materialize for the 0° grain orientation case, nor for other performance metrics for the 60° grain orientation case either. With all things considered, we believe it is preferable to use the original gray images for the purpose of damage prediction.

## Discussion

In this study, we applied a ConvLSTM-based neural network model for spatiotemporal representation learning. By feeding image sequences of stress and image sequences of damage to the model, we were able to train the model within 36 min and to generate a prediction on the next image of damage with good fidelity within 2 s. With this capability, out of every five images, one only needs to run the expensive PF simulation four times, generating the four input images, while the DL model would produce the fifth one. Roughly speaking, this saves 20% of the simulation cost. If used on physical data, such capability can be used for anticipating how a crack propagates in material (like batteries) health monitoring.

The achieved results show exceptional promise. Even for a low-resolution image, the output dimension/degrees of freedom are very high, making the video frame prediction problem challenging from a ML standpoint. Increasing the size of training data by adding more videos and considering a higher temporal resolution of the crack propagation process, which would effectively increase the training sample size, will likely enhance training. We do caution that the current predictive model is trained on a limited amount of data. Expanding the training dataset will undoubtedly increase both the robustness and stability of the model under different circumstances. In other words, based only on the current results, re-training will still be needed for different notch geometries.

One extension is to increase the capability in multiple-step ahead prediction. What is reported in this paper is one-step ahead prediction, which represents the current state of the art. Undoubtedly, more useful solutions would come from the ability to anticipate multiple steps ahead by feeding the generated frames back into the inputs and this will be the focus of future research. This may be further facilitated by integrating physical laws to a given boundary value problem and involving physics-informed neural networks in our method.^36^^,^^37^ Another extension is to acquire a suitable representation (low-dimensional embedding) for the crack growth process. The low-dimensional embedding would improve the sample efficiency of the training process and enable the system to discover a more complex growth process. Such crack prediction and connection to electrochemical signatures meet a significant need for the design of physics-based dynamic derating protocols for the analysis and proactive management of battery state-of-health.

### Limitations of the study

We introduce a DL model that approximates a segment of a multi-physics PF simulation video sequence in order to give an early-failure detection method. Due to the limited amount of simulation data available, this model was trained using a single pre-cut shape with a discrete number of preferential directions for the crack, which has various drawbacks.

Firstly, the simulation video does not contain any crystallographic defect that could affect the start and development of cracks in real materials. For example, stress accumulation may occur at different locations rather than only in the pre-notch tip area. It is necessary to adjust the input video or network structure specifically to mitigate the impact of noise and identify the critical cracking area. Secondly, the metrics in this study are also tailored to the simulation data. In the real microscopy video, crack branching can occur, and the damage zone is no longer diffused. It is essential to introduce a new metric to quantify the generated predictions to address this issue. Lastly, the preferential directions should be considered more explicitly to facilitate the predictions. Given that the crack length and growth pace vary depending on the direction, it is important to comprehend how these directions affect the stress/damage field both spatially and temporally.

## Resource availability

### Lead contact

Further information and requests for resources should be directed to and will be fulfilled by the lead contact, Sarbajit Banerjee (banerjee@chem.tamu.edu).

### Materials availability

This study did not generate new unique reagents.

### Data and code availability

• All data reported in this paper will be shared by the lead contact upon request.
• All original code has been deposited at Zenodo and is publicly available as of the date of publication. DOIs are listed in the key resources table.
• Any additional information required to reanalyze the data reported in this paper is available from the lead contact upon request.

## Acknowledgments

We acknowledge support from the 10.13039/100000001National Science Foundation (NSF) Award CMMI2038625 as part of the NSF/DHS/DOT/NIH/USDA-NIFA Cyber-Physical Systems Program and CNS-2328395 as part of the Future Manufacturing program. We further acknowledge support of crystal growth under NSF DMR 1627197.

## Author contributions

Under the supervision of S.B. and Y.D., Q.Z. designed the deep learning models and evaluated their performance. S.R. and B.-X.X. provided the phase-field simulation data and interpretation concepts. L.C. and R.D. provided experimental data, theoretical clarification of chemo-mechanics processes, and design ideas for the model. Each author discussed the findings and contributed to the final manuscript draft.

## Declaration of interests

The authors declare no competing interests.

## STAR★Methods

### Key resources table

**Table:** 

REAGENT or RESOURCE	SOURCE	IDENTIFIER
**Chemicals**		

Lithium hydroxide	Sigma Aldritch	Prod#: 920312
Vanadium (V) oxide	Sigma Aldritch	Prod#: 221899
Ethanol	Sigma Aldritch	Cat#: EX0276-3
Nitrosonium Tetrafluorborate	Alfa Aesar	Cat#: A15806-18
Acetonitrile	Sigma Aldritch	Cat#: EM-AX0143-6

**Deposited data**		

Code for deep-learning-based modeling	This paper	Zenodo: https://doi.org/10.5281/zenodo.12668274

**Software and algorithms**		

Spyder 4.2.5	Open source	https://www.spyder-ide.org/
TensorFlow 2.9.1	Open source	https://www.tensorflow.org/
OpenCV 4.6.0.66	Intel	https://opencv.org/

### Method details

#### A short review on chemistry-mechanics

Chemistry-mechanics coupling in materials encompasses the interplay between mechanical, chemical, and electric-field-driven forces during principal electrochemical processes.^38^^,^^39^ A considerable amount of attention has focused on the repercussions arising from the coupling of these electrochemical and mechanical processes.^20^^,^^40^^,^^41^^,^^42^ In typically brittle positive electrode materials, chemo-mechanical phenomena are particularly of significance in materials possessing multiple intercalation-induced phase transformations.^43^ These coexisting phase transformations can lead to cracking and particle degradation regimes specifically along the phase boundaries.^44^ Furthermore, particle degradation phenomena compound across length scales and can result ultimately in large-scale pulverization of the material and resultant loss of capacity.^29^

Insertion and deinsertion of Li-ions within positive electrode materials during discharging and charging regimes drive structural phase transformations, substantial dilation/contraction of crystal lattices, and thus engender directional stresses.^1^ Li-ion insertion in 2D materials leads to weakened van der Waals’ interactions between layers, altered layered stacking, and plane slippage.^45^ Furthermore, heterogeneous lithiation within single particles drives inhomogeneous deformations and lattice incommensurability at the solid-solid interface between phase boundaries and gives rise to compounding stresses that begin at the atomistic level and scale to the electrode level, resulting in fracture, dislocations,^7^ and delamination.^40^ Ceramic cathodes, such as Li_x_V_2_O_5_, are prone to damage regimes upon intercalation after only a few cycles, even when they only undergo small volume changes (ca. 2–8%), due to their characteristically brittle nature^44^^,^^45^^,^^46^^,^^47^^,^^48^^,^^49^^.^ Nucleation and growth regimes of microcracks oftentimes result from the initiation and accumulation of misfit dislocations.^50^ Such formation of new surfaces leads to altered diffusion pathways and amplifies lithiation heterogeneities with their accompanied stresses.^51^^,^^52^

Gaining understanding of and having the ability to predict chemo-mechanical processes occurring in electrode materials is essential toward informing design of cathode materials and can further be leveraged to extend battery life and prevent battery failure in commercially deployed systems by informing battery monitoring and derating algorithms.^29^ Battery monitoring systems which actively monitor the health of the battery for signs of thermal-runaway-triggering processes are crucial for mitigating catastrophic failure.^53^^,^^54^ Most of the methods implemented rely upon electrochemical metrics such as measurement of the anode overpotential or detection of characteristic high voltage plateau upon discharge to detect the presence of plated metallic lithium on non-metallic anodes. These metrics can be used to mitigate failure by triggering a response such as a reduction in the rate of charge.^55^^,^^56^ Measurement of proxies of chemo-mechanical degradation are less developed. Deciphering electrochemical signatures of mechanical failure mechanisms as is the focus of this work could indeed provide a valuable method to monitor degradation.

Derating strategies represent a complementary idea, whereby battery operating windows are narrowed across the lifetime of use allowing manufacturers to achieve the greatest balance possible between optimizing performance versus ensuring safety and longevity.^57^ Derating strategies can be static, where limits set for metrics such as system temperature, state of charge, voltage, or resistance serve as cut-off points for derating processes to initiate, resulting in a corrective response such as lowering the current density or preventing further charge/discharge. Dynamic derating strategies can alternatively allow for adaptive changes in the cut-off points based on the state of health of the battery system approximated using metrics such as capacity loss or increases in resistance. A major challenge is that cut-off limits are typically set based on empirical relationships established between the effects of using different derating metric cut-off points versus the resulting extension of battery life. Establishing more generalizable models will require building data-enabled frameworks which are informed by decoupled and direct measurements of the primary degradation processes. Understanding the likelihood of crack formation and propagation mechanisms is important for the decoupling or direct measurement of degradation.

#### SEM phase-field model

It is assumed that the bulk undergoes elastic deformation together with a quasi-brittle fracture at small deformation. The material volume changes due to the concentration field captured by the additional chemical strain tensor $\varepsilon_{c}=\left(c-c_{0}\right)\Omega$. Here, c is the Li concentration, and $c_{0}$ is the initial concentration. The total strain tensor $\varepsilon =\frac{1}{2}\left(\nabla u+\nabla u^{T}\right)=\varepsilon_{e}+\varepsilon_{c}$, is additively decomposed to elastic $\varepsilon_{e}$ and chemical parts $\varepsilon_{c}$. The total energy is divided into the elastic part $\psi_{e}$, chemical part, $\psi_{c}$ and damage part $\psi_{d}$ :

(Equation 5) $$\psi \left(\varepsilon ,c,\nabla c,d,\nabla d\right)=\psi_{e}\left(\varepsilon ,c,d\right)+\psi_{d}\left(c,d,\nabla d\right)+\psi_{c}\left(c,\nabla c\right)$$

The elastic energy is given by:

(Equation 6) $$\psi_{e}=\frac{1}{2}\varepsilon_{e}∶C\left(c,d\right)∶\varepsilon_{e}=\frac{1}{2}\left(\varepsilon -\varepsilon_{c}\right)∶\left(f_{d}C_{0}\left(c\right)+\left(1-f_{d}\right)P\right):\left(\varepsilon -\varepsilon_{c}\right)$$

The stiffness tensor is disaggregated through a damage function $f_{d}\left(d\right)=\frac{{\left(1-d\right)}^{2}}{{\left(1-d\right)}^{2}+a_{1}d\left(1+a_{2}d\right)}$ and $a_{1}=\frac{4EG_{c}}{\pi l_{c}\sigma_{u}^{2}}$, $a_{2}=-0.5$^13^^,^^14^. Moreover, $C_{0}$ is the undamaged elasticity tensor and to avoid cracking in the compressive regime, the tensor P is introduced in Amor et al.^11^ In phase-field damage models, the crack surfaces in the bulk are replaced with a diffusive damage zone and the fracture energy is dissipated via a crack density function γ^12^^,^^13^^,^^14^:

(Equation 7) $$\psi_{d}=G_{c}\left(c\right)\gamma \left(d,\nabla d\right)=\frac{G_{c}}{\omega_{0}}\left(\frac{1}{l_{c}}\omega \left(d\right)+l_{c}\nabla d\cdot A\cdot \nabla d\right)$$

Here, $\omega \left(d\right)=2d-d^{2}$ and the internal length scale parameter is denoted by $l_{c}$. The constant scaling parameter $\omega_{0}=4\int_{0}^{1}\sqrt{\omega \left(d\right)}dd=\pi$. Finally, the second-order structural tensor A=I+αa⨂a is constructed based on the vector $a={\left[\cos\left(\phi \right)\sin\;\left(\phi \right)\right]}^{T}$. Utilizing a non-zero value for the parameter α, one can penalize the crack direction along the angle ϕ^13^. The angle ϕ is treated as a constant input parameter and is in accordance with the preferential crack direction.

The chemical energy within the bulk of the material is given by:

(Equation 8) $$\psi_{e}=RTc_{\max}\left[\tilde{c}\ln\tilde{c}+\left(1-\tilde{c}\right)\ln\;\left(1-\tilde{c}\right)\right]+RTc_{\max}\chi \tilde{c}\left(1-\tilde{c}\right)$$

Here, $\tilde{c}=c/c_{\max}$ is the normalized concentration, R is the gas constant, T is the reference temperature, and κ is an interphase parameter. The first term in Equation 8 represents the entropic contribution to the system and the second term stands for the enthalpic contribution, which favors the separation of the system.

For the thermodynamic forces we have^8^^,^^10^:

(Equation 9) $$\sigma =\frac{\partial \psi }{\partial \varepsilon }=\frac{\partial \psi_{e}}{\partial \varepsilon }=C\left(d\right)∶\left(\varepsilon -\varepsilon_{c}\right)=C\left(c,d\right)∶\varepsilon_{e}$$

(Equation 10) $$\mu =\frac{\partial \psi }{\partial c}=RT\left(\ln\frac{\tilde{c}}{1-\tilde{c}}\right)+\frac{1}{2}\varepsilon_{e}∶\frac{\partial C}{\partial c}∶\varepsilon_{e}-\sigma ∶\Omega$$

(Equation 11) $$Y=\frac{\partial \psi }{\partial d}=\frac{\partial \left(\psi_{e}+\psi_{d}\right)}{\partial d}=\frac{1}{2}\varepsilon_{e}∶\frac{\partial C}{\partial d}∶\varepsilon_{e}+G_{c}\frac{\omega^{\prime }\left(d\right)}{\pi l_{c}}$$

(Equation 12) $$H=\frac{\partial \psi }{\partial \nabla d}=\frac{\partial \psi_{d}}{\partial \nabla d}=\frac{2}{\pi }G_{c}l_{c}\nabla d$$

(Equation 13) $$J=-M\left(c,d\right)\nabla \mu =-h_{d}\left(d\right)M_{0}\left(c\right)\nabla \left(RT\;\ln\left(\frac{\tilde{c}}{1-\tilde{c}}\right)-\sigma ∶\Omega \right)$$

A summary of governing equations for displacement, concentration, as well as damage field, is provided in Table 2. For the displacement and damage field we apply a variational derivative with respect to u and d to obtain the relative differential equation.

**Table 2:** Chemo-mechanical coupled formulation of cohesive fracture

Displacement	Concentration	Damage
∇·σ+b=0	$\nabla \cdot J+\dot{c}=0$	∇·H+Y=0
$\sigma =C\left(d\right)∶\varepsilon_{e}$	J=−M(c,d)∇μ	$H=\frac{2l_{c}}{\pi }G_{c}\left(c\right)\nabla d$
$\varepsilon_{e}=\varepsilon -\left(c-c_{0}\right)\Omega$	$\mu =\mu_{net}-\nabla \cdot \zeta$	$Y=-f_{d}^{\prime }H-\omega^{\prime }\frac{G_{c}}{\pi l_{c}}$

In Table 2, the expression H is defined as the maximum value between the undamaged elastic strain through the simulation time $\psi_{e}^{0}\left(t\right)=\frac{1}{2}\varepsilon_{e}∶C_{h}∶\varepsilon_{e}$, and the damage energy threshold $\psi_{th}=\frac{\sigma_{u}^{2}}{2E}$, i.e., we have $H=\max_{t}\;\left(\psi_{e}^{0}\left(t\right),\psi_{th}\right)$^13^.

#### Deep learning model

**Figure 8:**
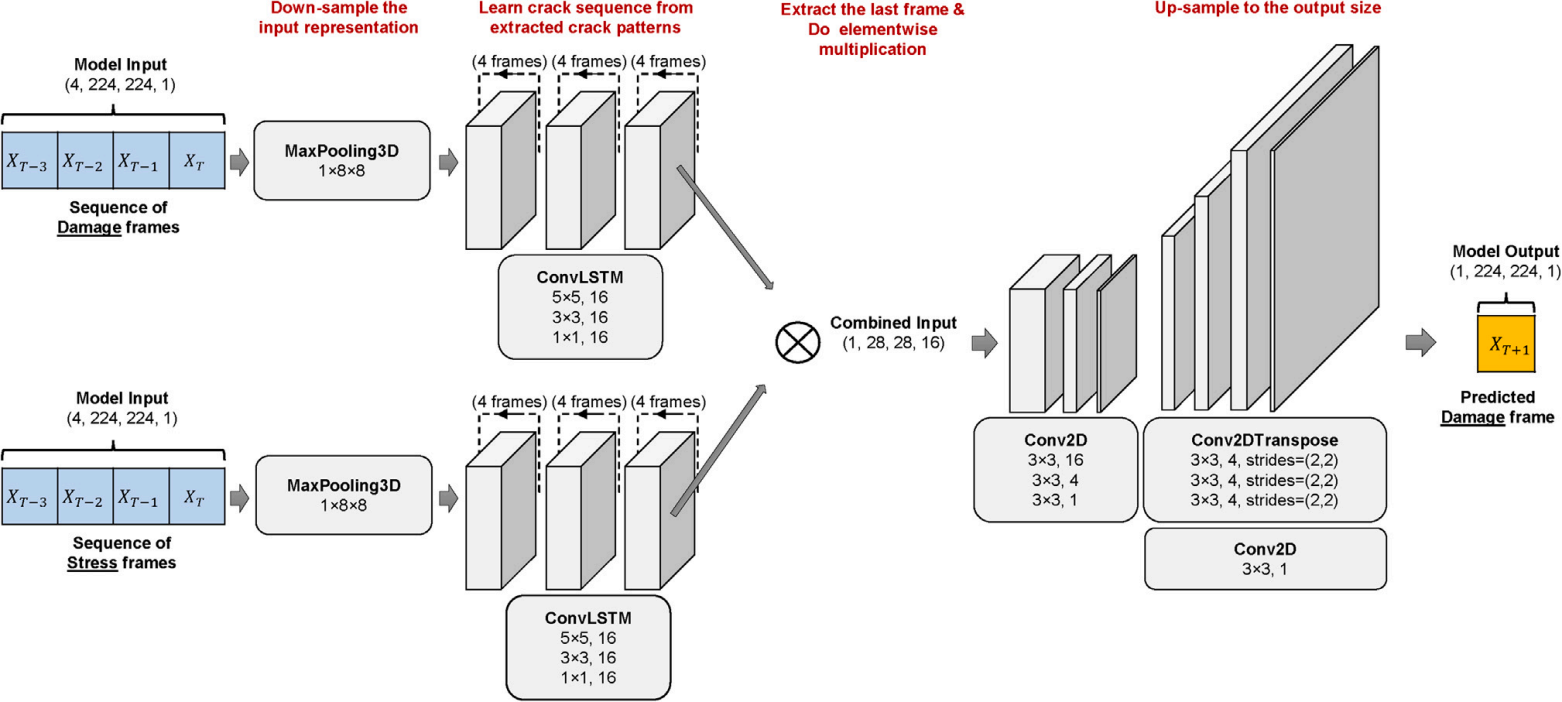
Layers in the ensemble model Damage and stress frames are respectively fed into the ConvLSTM network for extraction of spatiotemporal information. The extracted information is then combined via pairwise multiplication and used to generate a prediction for the next frame.

A collection of images or a video can be used to simulate and represent the evolution of a particle’s stress and damage. The primary objective of the deep learning model is to predict the future state of a particle based on its historical images of stress and damage. The results shown in this study are obtained by two different deep learning models, the ensemble model and the damage model, with the primary distinction being whether or not the stress images are included as inputs. The ensemble model’s architecture and layer parameters are shown in Figure 8. The damage model deploys only the top segment of the ensemble model. Its structure and layer parameters can be seen in Figure S1.

For both models, we apply downsampling and max pooling to the input frames. These operations enhance the ability of translation invariance for the resulting model and reduce the size of the image to make the image representation manageable. Translation invariance means that a small translation in the images does not significantly affect the outcomes of the model. Then, given a sequence of input images, we employ the ConvLSTM-based model to capture the temporal relationships underlying crack propagation. The final frame of the input sequence, which contains the spatial and temporal information of the entire sequence, is extracted by a subsequent convolutional network for prediction. For the damage model, this final frame contains only damage information, whereas for the ensemble model, the last frame of the damage input and the stress input are multiplied element-by-element to produce a new frame. For both models, the subsequent convolutional network is identical, i.e., using some 2D convolution and deconvolution layers to predict the damage at time t + 1 after going through an upsampling process. We use ConvLSTM as our choice of deep learning network with a ReLU activation function for the hidden layers. The code is implemented using Python libraries Keras^58^ and Tensorflow.^59^ We select the mean squared error as the loss function and train the neural network model for one hundred iterations with a batch size of five. To prevent overfitting, the order of training data is shuffled before every epoch. The Adam optimizer is used to minimize the loss and update the weight matrices in the network. The learning rate is 0.001. The training process costs around 36 min on a single A100 40GB GPU.

#### Materials

Reagents and their commercial sources are as follows: LiOH (Sigma Aldrich, 99.9%), V_2_O_5_ (Sigma Aldrich, 99.6%), ethanol (Sigma Aldrich, ≥99.6%), NOBF_4_ (Alfa Aesar, 98%), acetonitrile (Sigma Aldrich, Drysolv ≥95%), finder grids (Ted Pella, Cu 200 mesh).

#### Synthesis of α-V_2_O_5_ single crystals

Single crystals were first synthesized as δ-Li_0.7_V_2_O_5_ powder using a solvothermal process. Stoichiometric amounts of LiOH (Sigma Aldrich, 99.9%) and V_2_O_5_ (Sigma Aldrich, 99.6%) and 86 mL of ethanol (Sigma Aldrich, ≥99.6%) were added to a PTFE-lined stainless-steel autoclave (Parr, 125 mL capacity) and allowed to react for 72 h at 210 °C. The resulting powder was filtered and allowed to dry overnight. The powder was ground and annealed at 600 °C in a tube furnace under a flow of Ar gas for 12 h to remove residual moisture. To obtain large crystals, the resulting powder was ball-milled again, sealed in a quartz ampoule under vacuum, then melted at 800 °C and cooled at a rate of 2 °C/h in a programmable furnace (Thermo Scientific, Lindberg Blue M with UT150 controller) to obtain large black single crystals.

To obtain α-V_2_O_5_ single crystals, topochemical deintercalation of δ-Li_0.7_V_2_O_5_ was performed by treating them with 1.5 M equivalents of NOBF_4_ (Alfa Aesar, 98%) in dry acetonitrile (ca. 0.01 M solution) (Sigma Aldrich, Drysolv ≥95%) for 24 h. The leaching of Li ions and oxidation of V^4+^ to V^5+^ caused a drastic change in color from lustrous black to yellow/orange single crystals. Furthermore, cracks were observed along blacks in a layer-like habit exhibiting the 2D nature of the thermodynamically stable V_2_O_5_ structure.

#### Scanning electron microscopy characterization

Large single crystals were ground using a mortar and pestle to yield smaller single crystals with lateral dimensions 100–200 μm were fixed to transmission electron microscopy finder grids (Ted Pella, Cu 200 mesh). The sample was then mounted to a scanning electron microscope focused-ion beam (FIB-SEM). FIB-SEM images were performed using a Tescan LYRA-3 equipped with a Schottky field emission electron source and fully integrated Canion Ga focused ion beam column. The instrument also contained a 5-resevoir gas injection system (GIS) with W, Pt, SiO_X_, H_2_O, and XeF_2_. The SEM functionality was solely used for the characterization within this manuscript. Low magnifications were initially used to raster the grid for ideal single crystals presenting many facets, sizes of 100–200 μm, and flat surfaces.

#### Simulation images

A simulation video sequence needs to be converted into a certain size of images before being fed into the deep learning model. The video capture technique in OpenCV is used to first extract the frames, which are then saved in the preferred color space. After that, unnecessary regions are clipped out, leaving only the damage/stress field, which is then resized to 224 × 224 pixels and prepared for training and testing the model.
