# Supplementary material for: Chemomechanical damage prediction from phase-field simulation video sequences using a deep-learning-based methodology
Source: iScience. 2024 Aug 26;27(9):110822. doi: 10.1016/j.isci.2024.110822 (PMC11416508; doi:10.1016/j.isci.2024.110822)
Supplement: Document S1. Figure S1 [file mmc1.pdf]

iScience, Volume *27*

Supplemental information

Chemomechanical damage prediction from phase-field simulation video sequences using a deep-learning-based methodology

Quan Zeng, Shahed Rezaei, Luis Carrillo, Rachel Davidson, Bai-Xiang Xu, Sarbajit Banerjee, and Yu Ding

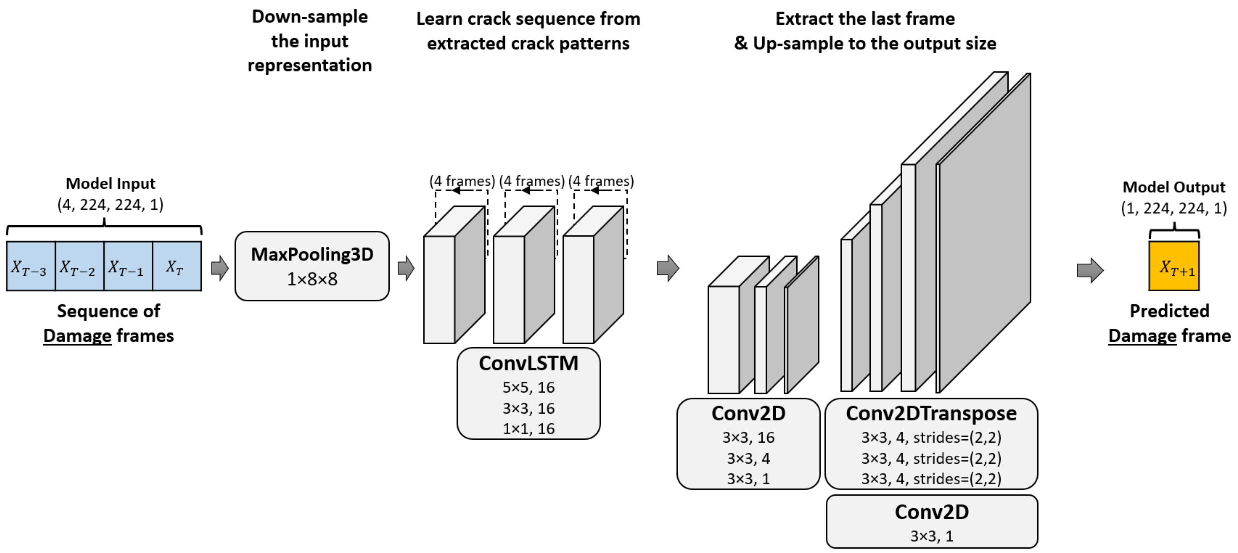

Figure S1. Layers in the damage model, related to Figure 8. Unlike the ensemble model, sequences of damage frames are the sole input for the damage model, and there is no pairwise multiplication operation between the damage and stress data.
